# Arabic-speaking migrants’ attitudes, opinions, preferences and past experiences concerning the use of interpreters in healthcare: a postal cross-sectional survey

**DOI:** 10.1186/1756-0500-7-71

**Published:** 2014-02-03

**Authors:** Emina Hadziabdic, Björn Albin, Katarina Hjelm

**Affiliations:** 1 School of Health and Caring Sciences, Faculty of Health and Life Sciences, Linnaeus University, SE-351 95, Växjö, Sweden

**Keywords:** Arabic-speaking migrants, Communication, Cross-sectional survey, Healthcare encounter, Structured self-administered questionnaire, Use of interpreters

## Abstract

**Background:**

Good communication is an important prerequisite for equal treatment in a healthcare encounter. One way to overcome language barriers when patients and healthcare staff do not share the same language is to use a professional interpreter. Few previous studies have been found investigating the use of interpreters, and just one previous study from the perspective of European migrants, which showed that they perceived interpreters as a communication aid and a guide in the healthcare system as regards information and practical matters. No previous study has gathered quantitative information to focus on non-European migrants’ attitudes to the use of interpreters in healthcare encounters. Thus, the aim of this study was to investigate Arabic-speaking individuals’ attitudes, opinions, preferences and past experiences concerning the use of interpreters in healthcare in order to: (i) understand how persons’ expectations and concerns regarding interpreters may vary, both within and across cultural/linguistic populations; (ii) understand the consequences of diverse opinions/expectations for planning responsive services; and (iii) confirm findings from previous qualitative studies.

**Method:**

A postal cross-sectional study using a structured self-administered 51-item questionnaire was used to describe and document aspects of Arabic-speaking individuals’ attitudes to the use of interpreters in healthcare. The sample of 53 Arabic-speaking migrants was recruited from three different places. Participants were mostly born in Iraq and had a high level of education and were almost equally divided between genders. Data were analysed with descriptive statistics.

**Results:**

The main findings were that most of the participants perceived the interpreter’s role as being a communication aid and a practical aid, interpreting literally and objectively. Trust in the professional interpreter was related to qualification as an interpreter and personal contact with face-to-face interaction. The qualities of the desired professional interpreter were: a good knowledge of languages and medical terminology, translation ability, and sharing the same origin, dialect and gender as the patient.

**Conclusion:**

This study confirmed previous qualitative findings from European migrant groups with a different cultural and linguistic background. The study supports the importance of planning a good interpretation situation in accordance with individuals’ desire, irrespective of the migrant’s linguistic and cultural background, and using interpreters who interpret literally and objectively, who are highly trained with language skills in medical terminology, and with a professional attitude to promote communication, thus increasing cost-effective, high-quality individualized healthcare.

## Background

Individualized and holistic healthcare is based on ideals of equal value and fair and equal treatment for all members of society [1]. One important prerequisite for equal treatment in a healthcare encounter between healthcare professionals and migrants is clear communication [2,3]. Unclear communication is associated with disparate healthcare access and healthcare outcomes for migrants [4,5]. One way to overcome barriers in communication between healthcare professionals and migrants is to use professional interpreters [6-8].

To our knowledge, no previous study has focused on non-European migrants’ attitudes, opinions, preferences and past experiences concerning the use of interpreters in healthcare encounters based on quantitative information. However, there are previous qualitative studies in Sweden examining how Bosnian/Croatian/Serbian-speaking migrants [9], healthcare staff [10] and family members [11] perceive the use of interpreters in healthcare. The mixture of immigration to Sweden has changed in recent years, with an increasing number of people from outside Europe [12]. Today the largest group coming to Sweden are Arabic-speaking migrants from the Middle East [13]. Due to these changes, it is important to investigate a larger population and a different migrant group with a different cultural and language background in order to: (i) understand how persons’ expectations and concerns regarding interpreters may vary, both within and across cultural/linguistic populations; (ii) understand the consequences of diverse opinions/expectations for planning responsive services; (iii) confirm findings from previous qualitative studies. However, migrants in Sweden represent 171 different nationalities and constitute a very heterogeneous group, with Arabic-speaking migrants as the largest group of migrants coming to Sweden in recent years, a trend that is expected to continue [14].

A literature search showed that European migrants speaking the Bosnian/Croatian/Serbian languages and living in Sweden perceived interpreters as a communication aid and a guide to the healthcare system as regards information and practical issues [9]. Furthermore, they desired an interpreter with good skills in medical terminology and language, and with a professional attitude in personal contact through face-to-face interaction. Another investigation [15] focusing on Bosnian/Croatian/Serbian and Russian refugees in Ireland showed that use of informal interpreters could be experienced as inadequate and problematic because patients were often left worried, frustrated with experiences of error and misdiagnosis and unsure about following doctors’ advice for treatment at the end of their consultation. Particularly migrants of Asian origin in the UK expected that a good interpreter should empathize with them and understand and relate to their situation [16]. Additionally, patients preferred to use family members as interpreters [16,17]. Differences in cultural background and lack of confidence in interpreters were seen as problems for cross-cultural communication for Kurdish war-wounded refugees in Sweden [18]. In summary, there is no knowledge of non-European, Arabic-speaking individuals’ attitudes, opinions, preferences and past experiences concerning the use of interpreters in healthcare to assist in promoting communication for individuals who use interpreters in order to develop an affordable and accessible system of healthcare encounters. Thus, the aim of this study was to investigate Arabic-speaking individuals’ attitudes, opinions, preferences and past experiences concerning the use of interpreters in healthcare.

## Method

### Design

A postal cross-sectional survey using a structured self-administered questionnaire [19] was used to describe and document aspects of Arabic-speaking individuals’ attitudes, opinions, preferences and past experiences concerning the use of interpreters in healthcare.

### Setting

Participants were recruited from three different cities in a county in Sweden, where 13.5% of the population was born abroad [20] and where interpreters are frequently use in diverse healthcare services.

People who have communication barriers are entitled to access to an interpreter in all contacts with healthcare service according to Swedish law [21] (for more details see [22]. In the studied area the responsibility for arranging an interpreter in a healthcare encounter lies with the healthcare service. The interpreter service agencies are often run by private enterprises outside health care. National guidelines for professional interpreters include literally translating, being neutral, ensuring confidentiality and not making any other statements unrelated to the situation [22].

### Participants and procedure

Criteria for inclusion in the study were Arabic-speaking adults (aged over 18) participants who use interpreters in healthcare, differing in gender, age, educational level and length of residence in Sweden.

To come into contact with participants the principal investigator contacted representatives of adult education facilities for immigrants by telephone. The representatives were requested to invite Arabic-speaking persons with different backgrounds living in Sweden to participate in an information meeting. A time was set for meetings when information (verbal and written) was given by the principal investigator (EH) about the aim of the study, focusing on the use of an interpreter in healthcare encounters from their perspective, about the implementation of the study and the ethical considerations. Written information and a questionnaire together with a prepaid envelope were given to voluntary participants so they could answer when appropriate. The questionnaire in the prepaid envelope was to be returned to the principal investigator. The principal investigator’s contact details were included in case the participants had any questions. Participants did not have to write their names if they did not want to.

Ninety questionnaires were distributed between December 2011 and February 2012 at three different adult education facilities for immigrants. In total 53 persons returned the questionnaire, giving a response rate of 52% (see Table 1). All were refugees, born mostly in Iraq, with a high level of education.

**Table 1:** Characteristics of the study population

**Variable**	**Arabic-speaking individuals (N = 53)**
Gender *(n)*	
Male	24
Female	23
Missing	6
Age (years)	
18–28 years	15
29–39 years	13
40–49 years	15
50–59 years	7
Missing	3
Length of residence in Sweden (years)*	9 (1–11 years)
Missing	5
Educational	
Primary school	5
Secondary school	16
University ≤ 2 years	12
University ≥ 2 years	8
Missing information	12

*Values are median (range).

### Data collection

A 51-item questionnaire with some additional background data questions was developed based on four previous qualitative studies [10,11,23,24] concerning the use of interpreters in healthcare to ensure content validity [19]. When formulating questions, the results from the previous qualitative studies were organized into three different areas: questions related to the individuals’ attitudes to the use of interpreters as a communication aid in healthcare (21 items); questions related to the individuals’ attitudes to the professional and personal qualities of an interpreter in healthcare (19 items); and questions related to the individuals’ attitudes to modes of interpretation and the types of interpreter in healthcare (11 items).

The questionnaire was first written in Swedish and then translated into Arabic by a professional translator and then back into Swedish by another professional translator [25]. In order to avoid communication misunderstandings, the questionnaire was pilot-tested by 20 Arabic-speaking persons. They found the questions in the questionnaire clear, concise and easy to answer. Thus, no changes were made and those respondents were included in the study.

Arabic-speaking persons responded to statements in the questionnaire by giving a rating on an ordinal 4-point Likert scale, ranging from 1 (strongly disagree) to 4 (strongly agree), i.e. the higher the values, the stronger the dimension of agreement. The questionnaire included three areas of perceiving the use of interpreters in healthcare: communication aid (21 items); the professional and personal qualities of an interpreter (19 items); modes of interpretation, and the type of interpreters (11 items). The data is presented as selected dichotomous variables, by summing responses strongly disagree and disagree respective strongly agree and agree in order to report as dichotomous variables as possible to show either a positive or negative view, since the participants mostly responded on only two values respectively by either disagreeing or agreeing.

### Data analysis

Descriptive statistics, in terms of frequencies and percentages, were used to analyse [19] the three areas of the use of interpreters with the help of SPSS version 20 (SPSS Inc, Chicago, IL, US). The 35 variables were chosen for presentation in the table in order to make the table more manageable and easy to read [26]. However, responses from all included 51 variables are reported in the results section. Thus, nothing has been omitted.

### Ethical considerations

The study followed Swedish law concerning the regulation of ethics in research involving humans [27] and has been conducted in accordance with established ethical principles for human clinical research in the guidelines stated in the World Medical Association’s Declaration of Helsinki [28]. Participants were given written information about the study, assured of confidentiality and the right to withdraw. To preserve the confidentiality of the participants’ data, the questionnaires were anonymous and coded by number. The analysis and presentation of the data was done on group level and in a way that concealed the participants’ identity. All the collected data were stored in a locked space accessed only by the principal investigator [27,28]. The procedure was in accordance with Swedish law [27] and approval by an official research ethics committee was not required as the research study posed no physical or mental risk to the participants and did not treat participants’ personal data.

## Results

### Communication aid

Almost all of the participants perceived an interpreter as a communication aid when people could not speak the same language (94%), translate information (90%) so that they could understand and make themselves understood (91%) (see Table 2). With few exceptions (96%) the participants perceived that, besides being a communication aid, the interpreter had an important role in helping them to find the right way to and within the healthcare system, as more than half of participants (67%) were unable to read signs.

**Table 2:** Questions concerning Arabic-speaking individuals’ attitudes to the use of interpreters as a communication aid in healthcare

**Variable**	**N**	**Agreed N (%)**	**Disagree N (%)**	**Mean ± Sd.**
It is important that the interpreter helps you to find the way within health care to different consultations because it is difficult for me to read signs in English	53	51 (96%)	1 (2%)	3.9 ± 0.4
The interpreter helps me only with translation because I do not speak Swedish	53	50 (94%)	3 (6%)	3.8 ± 0.6
While I talk through an interpreter it is important to express myself clearly so the interpreter will be able to support me	52	48 (91%)	4 (8%)	3.7 ± 0.7
A nurse/physician always books an interpreter in advance when I need it	52	47 (87%)	5 (10%)	3.5 ± 0.7
It is difficult to guarantee that what I have said during the interpretation is made in confidence and that the interpreter will not spread it to others	53	45 (85%)	8 (15%)	3.3 ± 0.8
I prefer an interpreter who helps me with transport both before and after consultations in healthcare	52	36 (67%)	16 (15%)	3.0 ± 1.1
I always get a feeling of uncertainty while I talk through an interpreter because I do not know whether what I say is correctly translated or not	53	32 (60%)	21 (40%)	2.6 ± 1.1
I find that talking through an interpreter reduces intimacy between healthcare staff and me	51	30 (57%)	21 (40%)	1.9 ± 1.0
Talking through an interpreter makes me feel handicapped as I cannot speak Swedish	53	23 (43%)	30 (57%)	2.3 ± 1.2
I have not been in any situations in healthcare when booked interpreters have not turned up	51	23 (43%)	28 (53%)	2.4 ± 1.2
I always get a feeling of uncertainty while I talk through an interpreter because I do not know whether what I say is correctly translated or not	53	15 (28%)	38 (72%)	1.9 ± 1.0
The interpreter should not interpret literally and objectively	52	12 (25%)	40 (75%)	1.7 ± 1.2

Three quarters of the participants (75%) thought that the interpreter should interpret literally without any value judgments being made.

Little more than a quarter of individuals (28%) perceived that talking through an interpreter gave a feeling of insecurity. Nearly half of participants (43%) perceived the use of an interpreter as a form of physical handicap. Furthermore, for more than half of participants (57%) the interpreters could decrease the intimacy in the relationship between individuals and healthcare staff; almost one third (30%) thought that the presence of an interpreter made them forget to say some things; and almost one third (29%) felt insecure when talking through interpreters, and almost half of participants (40%) concerning the sensitive matters especially. More than half of the participants (60%) (19 men and 6 women) felt insecure as to whether or not interpreters were able to translate what they said literally. Over three quarters (85%) also felt uncertain about the interpreter’s respect for confidentiality. Almost three quarters of the participants (72%) expressed that room where interpretation occurs are significant. The majority of the respondents (83%) felt that a secluded room for an interpretation situation was an important factor to be able to understand and feel safe. However, many of the participants (68%) did not think that continuous use of the same interpreter was important.

It was found that healthcare staff booked interpreters for the majority of respondents (87%) However, little more than half of participants (53%) had experienced situations in healthcare when no interpreter had turned up for the appointed consultation. Participants thought that it was important that healthcare staff book an interpreter for every consultation (70%), with the possibility of replacing a particular interpreter if patients did not feel trust (98%).

### The professional and personal qualities of an interpreter

Most participants (94%) thought that feeling trust in an interpreter is essential for whether they openly talk about their health condition in a healthcare encounter. The professional interpreter’s language skill in both languages, Swedish and Arabic, was rated as unimportant for almost all respondents (96%) (see Table 3). The professional interpreter’s religion was likewise of no importance for almost two thirds (66%). However, knowledge of medical terminology and translation ability were perceived as important for all informants, with few exceptions (94% and 96%). It was also important to three quarters of the participants (75%) that an interpreter should talk the same dialect as the individual, and having an interpreter trained in the language and having skill in medical terminology was important for most of the participants (83%).

**Table 3:** Questions related to the professional and personal qualities of an interpreter in the survey of Arabic-speaking individuals’ attitudes to the use of interpreters in healthcare

**Variable**	**N**	**Agree N (%)**	**Disagree N (%)**	**Mean ± Sd.**
It is of no importance whether an interpreter is fluent in both languages	52	51 (96%)	1 (2%)	3.9 ± 0.4
It is important that an interpreter has a great ability to translate	52	51(96%)	1 (2%)	3.9 ± 0.3
An interpreter should show me respect	53	51 (96%)	2 (4%)	3.8 ± 0.5
It is important that an interpreter have training both in the language and the terminology used in healthcare	52	50 (94%)	2 (3%)	3.9 ± 0.5
It is important that the interpreter is neutral and impartial	51	48 (91%)	3 (9%)	3.7 ± 0.6
The interpreter’s age is of no importance for the translation	52	41 (77%)	11 (21%)	3.1 ± 1.0
It is important that an interpreter talks the same dialect as me	53	40 (75%)	13 (25%)	3.3 ± 1.0
It is not important what clothes an interpreter wears and whether he/she is provocatively dressed	52	36 (68%)	16 (30%)	3.0 ± 1.3
It is not important what religion the interpreter belongs to	53	35 (66%)	18 (34%)	2.7 ± 1.2
It is important that I know what country the interpreter comes from	53	34 (64%)	19 (36%)	2.9 ± 1.2
I think that it is important to use an interpreter of the same gender as myself	51	31 (58%)	20 (38%)	2.8 ± 1.2
It is not important that the interpreter introduces him/herself to me before starting the interpretation session	53	16 (30%)	37 (70%)	2.0 ± 1.2
It is not important that an interpreter is trained	52	14 (26%)	38 (72%)	1.9 ± 1.1
It is of no importance to me whether the interpreter tells other people about what I have told the physician or nurse during my consultation in which he/she has interpreted	53	8 (15%)	45 (85%)	1.4 ± 0.9

One of the professional qualities, the education of interpreters, was important for almost three quarters of participants (72%). Other essential professional qualities included the interpreter showing respect (96%) for the parties involved in the encounter, introducing themselves (70%), maintaining the code of confidentiality (85%) and showing objectivity (91%).

Nearly two thirds of the participants (64%), mostly men (n = 21), found it important that the patient and the interpreter should share the same origin. More than half of the participants (58%) thought it essential that the interpreter should be of the same gender as the person needing the interpreter. To feel trust in the interpreters, it was important that an interpreter has similar outfit as the participant for almost half of the respondents (42%). However, interpreters wearing of non-provocative or neutral clothes and similar clothes to the participants’, and their age (with the exception of a few participants (17%) who felt trust in younger interpreters), were not important to nearly three thirds (68%) and three quarters (77%) of the respondents respectively.

### Modes of interpretation and the types of interpreter

The majority of the respondents (87%) preferred personal contact with face-to-face interaction with the interpreter, while nearly two thirds (64%) perceived no differences between face-to-face and telephone interpretation, as the opportunity to follow the interpreter’s body language was rated as not important by nearly two thirds of the participants (64%) (see Table 4). However, most participants (92%) preferred interpretation by telephone when discussing sensitive matters. The results showed that almost all individuals (98%) preferred to use a professional interpreter because of their professional training, ensuring high-quality language skills, and being employed by an interpreter agency.

**Table 4:** Questions related to modes of interpretation and the types of interpreter in the survey of Arabic-speaking individuals’ attitudes to the use of interpreters in healthcare

**Variable**	**N**	**Agree N (%)**	**Disagree N (%)**	**Mean ± Sd.**
It is good to use an interpreter who has special training and who is employed by an agency	53	52 (98%)	1 (2%)	3.8 ± 0.5
I prefer to use a telephone interpreter during sensitive investigations	52	49 (92%)	3 (8%)	3.7 ± 0.7
I prefer to use an interpreter in place	52	46 (87%)	6 (13%)	3.3 ± 0.8
Bilingual healthcare staff are good to use as interpreters because they are already in place when interpreting is to be done	49	46 (87%)	3 (13%)	3.5 ± 0.7
There is no difference between using telephone interpreters and interpreters in place	52	34(64%)	18 (36%)	2.6 ± 1.1
Using a family member/friend as interpreter implies that I get support from family/friend at the same time as he/she translates	52	33 (62%)	19 (38%)	2.7 ± 1.1
I feel confidence in using a family member/friend as an interpreter more than an unknown person being an interpreter	52	28 (53%)	24 (47%)	2.5 ± 1.2
I prefer to use a family member/friend as an interpreter	53	25 (47%)	28 (53%)	2.4 ± 1.1
There is no risk that all information will not be translated when I use a family member/friend as interpreter	51	17 (32%)	34 (68%)	2.0 ± 1.1

Nearly half of the participants (47%) wanted to use family members as interpreters. More than half of the respondents (62%) could see advantages in using family members as interpreters because they also provided support. Half of the participants (53%) expressed trust in family members as interpreters rather than an unknown interpreter. However, half of the informants (68%) could see the risk in family members as interpreters, on account of their inability to fully grasp the language.

Concerning the use of bilingual healthcare professionals as interpreters, positive experiences were reported as they gave fast and effective help without delay for the majority of individuals (87%). However, half of participants (51%) could see the risk in bilingual healthcare professionals as interpreters, on account of their inability to fully grasp the language.

## Discussion

This study is, to our knowledge, the first study to investigate a non-European migrant group, Arabic-speaking individuals, to elicit their attitudes, opinions, preferences and past experiences concerning the use of interpreters in healthcare. The main study findings document that participants preferred a face-to-face professional interpreter who was an objective aid in communication and practical matters, with a good knowledge of medical terminology, good translation ability and sharing the same origin, dialect and gender as them. This study was an empirical quantitative study that can confirm knowledge from previous qualitative studies of European migrants speaking Bosnian/Croatian/Serbian [10,11,24,29].

A strength of this study could be that the participants were recruited from three different cities in a geographically defined area and healthcare institutions with similar organizations of interpreter services; they differed in age, length of residence in Sweden and educational level. However, they had similar migrational and socioeconomic status, were almost equally divided between genders and born mostly in Iraq. The demographic background in this study ensured a typical picture of the Arabic-speaking population in Sweden [30]. It is difficult to get persons to complete a questionnaire [19], so the response rate (52%) is fairly good for a questionnaire distributed without reminders being sent.

Developing a new, structured self-administered questionnaire in another language than the author’s must be noted as a limitation in this investigation. However, the translation of data was done by a professional translator and then back-translated into Swedish by another professional translator [25]. Finally, the questionnaire was pilot-tested by Arabic-speaking persons for accuracy in the transcriptions; still, the new structured self-administered questionnaire should be translated and developed in other languages to ensure its relevance. The study results strengthen the findings from previous qualitative studies of a different language group. Yet, it would need a further study with a larger sample to be able to generalize the study results.

The preferred mode of interpretation in healthcare encounters was face-to-face interaction with professional interpreters. This outcome supports those of previous qualitative studies from the perspective of Bosnian/Croatian/Serbian-speaking migrants living in Sweden [24] and Bosnian/Croatian/Serbian- and Russian-speaking migrants living in Ireland [15]. Documented benefits of the use of professional interpreters were that they significantly reduce errors with potential clinical consequences compared to an informal interpreter (family member or bilingual healthcare staff) [31] and the professional interpreter improves communication between patient and healthcare staff [6-8], which leads to better quality of treatment [32].

On the other hand, this study’s results are in contrast to those of previous qualitative studies involving mostly Asian-born respondents in the UK who preferred family members as interpreters [16,17]. The differences between the studies can be explained by the dissimilarity in the countries’ legal rights to interpreters and in their migration history. First, legislation to enforce support through interpretation is in effect in Sweden [33] but unclear or lacking in other countries including the UK [34]. Previous studies in the UK [35] and the US [36] found that patients were unaware that healthcare staff could access interpreting services for their healthcare consultations. Second, Irish [37] and Swedish [12] migration populations consist mostly of labour migrants and forced migrants, and both Sweden and Ireland lack established migrant communities. In the UK [38], by contrast, there is mostly voluntary migration and the country has more established homogeneous migrant communities related to the Commonwealth. However, our findings may be relevant for newly arrived migrants in undeveloped migrant communities in countries that have a clear legal right to interpreters in healthcare encounters.

Confidence between parties in interpreter consultations is essential for effective communication and providing quality care [39,40]. This investigation showed that trust in the interpreter was associated with language skill in medical terminology, education, sharing the same dialect, showing respect and objectivity, and keeping the code of confidentiality. The qualities of interpreters discussed by participants were mostly in agreement with what has previously been stated by patients [16,24], healthcare staff [10] and family members [11]. The dissimilarities between studies were revealed in the fact that (i) the interpreter’s skill in both languages, (ii) the interpreter’s religion, (iii) the interpreter wearing neutral clothes and (iv) being able to follow the interpreter’s body language were not important factors for feeling confidence in the process. The dissimilarities and the opinions contradictory to this result might be due either to differences in the method used for investigation or else participants did not properly read the survey/question because the statement was negatively formulated. More studies are needed to investigate whether this may be due to the method of investigation or to the construction of the questionnaires.

A new finding compared to the qualitative studies among European, Bosnian/Croatian/Serbian-speaking, migrants living in Sweden [24] and family members [11] was that the majority of respondents experienced a willingness among healthcare professionals to book an interpreter when needed. One possible explanation for the differences between the studies could be the differences in the methods used. Another possible explanation could be that culture has an impact on how people respond to health and illness, which affects self-care, health-seeking behavior and health [2]. A study investigating beliefs about health, illness and healthcare in female Arabic-speaking migrants with gestational diabetes showed an active information-seeking behavior, in contrast to female Bosnian/Croatian/Serbian-speaking migrants with diabetes mellitus, who revealed a passive attitude to self-care, relying on healthcare staff for help [41]. In this study Arabic-speaking migrants might be required among healthcare staff to book interpreters, in contrast to Bosnian/Croatian/Serbian-speaking migrants who relied on the willingness of healthcare staff. A third explanation could be either the healthcare staff’s attitude to different migrant groups or healthcare staff’s increased knowledge and positive attitude over time between studies focusing on the importance of professional interpreters in healthcare encounters. A previous study [42] investigating healthcare professionals’ perceptions of beliefs about health and illness in migrants with diabetes mellitus found that a common attitude among healthcare staff to non-European migrants was that there were great dissimilarities in language and culture [42]. However, healthcare staff’s attitudes to migrant groups are important to consider in healthcare encounters, which in turn may influence the use of interpreters. The main goal in healthcare is individualized and holistic healthcare based on ideals of equal value and fair and equal treatment for all people [1].

## Conclusion

This investigation corroborated previous qualitative findings from another migrant group with a different cultural and linguistic background. Furthermore, this study confirms the complexity inherent in healthcare interpretation services. Similarities were found in the desire to have a face-to-face professional interpreter who was an objective aid to communication and practical matters, with a good knowledge of medical terminology, good translation ability and sharing the same origin, dialect and gender as them. Attitudes differed as to whether the interpreter should be equally competent in both languages, be of the same religion, wear neutral clothes, and as regards the willingness of healthcare professionals to book an interpreter.

The implication of the study is the importance of planning for the use of interpreters in accordance with the individuals’ desire, irrespective of the migrant’s linguistic and cultural background. It is also necessary for healthcare staff to put demands on interpreting agencies to offer neutral interpreters highly trained in medical terminology, and with a professional attitude in order to promote the use of interpreters in a healthcare encounter. Further, the use of interpreters in accordance with individuals’ desire can prevent and limit poor communication, thereby increasing cost-effective, high-quality individualized healthcare.

## Competing interests

The authors declare that they have no competing interests.

## Authors’ contributions

EH was responsible for the study conception and design, development of the questionnaire, carried out the data collection, performed descriptive statistical analyses and drafted and revised the manuscript. BA helped in the descriptive statistical analyses. BA and KH helped in the design of the study and coordination. EH, BA and KH performed the drafting of the manuscript. All authors read and approved the final manuscript.
